# Identifying patients with acute ischemic stroke within a 6-h window for the treatment of endovascular thrombectomy using deep learning and perfusion imaging

**DOI:** 10.3389/fmed.2023.1085437

**Published:** 2023-02-22

**Authors:** Hongyu Gao, Yueyan Bian, Gen Cheng, Huan Yu, Yuze Cao, Huixue Zhang, Jianjian Wang, Qian Li, Qi Yang, Lihua Wang

**Affiliations:** ^1^Department of Neurology, The Second Affiliated Hospital, Harbin Medical University, Harbin, Heilongjiang, China; ^2^Department of Radiology, Beijing Chaoyang Hospital, Capital Medical University, Beijing, China; ^3^Neusoft Medical System Co., Beijing, China; ^4^Department of Radiology, Liangxiang Teaching Hospital, Capital Medical University, Beijing, China; ^5^Department of Neurology, Peking Union Medical College Hospital, Peking Union Medical College and Chinese Academy of Medical Sciences, Beijing, China

**Keywords:** acute ischemic stroke, endovascular thrombectomy, stroke onset time, deep learning, perfusion imaging

## Abstract

**Introduction:**

It is critical to identify the stroke onset time of patients with acute ischemic stroke (AIS) for the treatment of endovascular thrombectomy (EVT). However, it is challenging to accurately ascertain this time for patients with wake-up stroke (WUS). The current study aimed to construct a deep learning approach based on computed tomography perfusion (CTP) or perfusion weighted imaging (PWI) to identify a 6-h window for patients with AIS for the treatment of EVT.

**Methods:**

We collected data from 377 patients with AIS, who were examined by CTP or PWI before making a treatment decision. Cerebral blood flow (CBF), time to maximum peak (Tmax), and a region of interest (ROI) mask were preprocessed from the CTP and PWI. We constructed the classifier based on a convolutional neural network (CNN), which was trained by CBF, Tmax, and ROI masks to identify patients with AIS within a 6-h window for the treatment of EVT. We compared the classification performance among a CNN, support vector machine (SVM), and random forest (RF) when trained by five different types of ROI masks. To assess the adaptability of the classifier of CNN for CTP and PWI, which were processed respectively from CTP and PWI groups.

**Results:**

Our results showed that the CNN classifier had a higher performance with an area under the curve (AUC) of 0.935, which was significantly higher than that of support vector machine (SVM) and random forest (RF) (*p* = 0.001 and *p* = 0.001, respectively). For the CNN classifier trained by different ROI masks, the best performance was trained by CBF, Tmax, and ROI masks of Tmax > 6 s. No significant difference was detected in the classification performance of the CNN between CTP and PWI (0.902 vs. 0.928; *p* = 0.557).

**Discussion:**

The CNN classifier trained by CBF, Tmax, and ROI masks of Tmax > 6 s had good performance in identifying patients with AIS within a 6-h window for the treatment of EVT. The current study indicates that the CNN model has potential to be used to accurately estimate the stroke onset time of patients with WUS.

## Introduction

In the guidelines for the early management of patients with acute ischemic stroke (AIS) published by the American Heart Association/American Stroke Association (AHA/ASA) in 2019, recombinant tissue-type plasminogen activator (rt-PA) thrombolysis and endovascular thrombectomy (EVT) are recommended to treat patients with AIS (1). Both of these are performed mainly for patients within a specific window of time from stroke onset, which are 4.5 h for rt-PA thrombolysis and 6-h for EVT. However, because 14–29.6% of patients with AIS are attacked during their sleep, which is called wake-up stroke (WUS) (2), their accurate stroke onset time cannot be ascertained to calculate this window. This means that other examinations are needed to estimate the stroke onset time of patients with WUS before treatment of rt-PA thrombolysis or EVT.

In previous studies, multi-modality imaging has been shown to have strong potential for accurately estimating the stroke onset time (3–8). In rt-PA thrombolysis treatment, the imaging biomarker of intensity mismatch between diffuse weighted imaging (DWI) and fluid-attenuated inversion recovery (FLAIR) is used to detect patients within a 4.5 h window (4), which means that the stroke onset time of patients with an unknown time and with a DWI–FLAIR mismatch biomarker is within a 4.5 h window for rt-PA thrombolysis treatment. In order to further explore the relationship between imaging biomarker and stroke onset time, Kong et al. constructed a decoder–encoder network to extract features using DWI, FLAIR, and time to maximum peak (Tmax) images, which can classify patients within a 4.5 h window for rt-PA thrombolysis treatment (8). This means that a machine learning classifier based on an imaging biomarker can accurately estimate the stroke onset time.

However, there is not a typical imaging biomarker to identify a 6-h treatment window for EVT. Some potential imaging biomarkers were found in previous works (9–12), such as a reduction in cerebral blood flow (CBF) and a delayed Tmax. The progression of AIS can be directly expressed by changes of an infarct core and ischemic region (12–14). An infarct core and penumbra region can be estimated using perfusion map images, which include CBF, cerebral blood volume (CBV), mean transit time (MTT), and Tmax. The infarct core is defined as the region of CBF reductions to < 30% compared contralateral hemispheres (CBF < 30%) for computed tomography perfusion (CTP), or apparent diffusion coefficient (ADC) values < 620. The ischemic region includes the infarct core and penumbra region, which is the region of Tmax > 6 s (9). Furthermore, Olivot et al. (15) estimated the benign hypoperfusion, ischemic, and infarct core regions only by different Tmax thresholds, which are, respectively, >4, >6, and >10 s. Thus, CBF and Tmax are significantly related to the stroke onset time.

The present study sought to combine the deep learning technique with perfusion map images (CBF and Tmax), which was processed from CTP or perfusion weighted imaging (PWI), to identify patients with AIS within a 6-h window for the treatment of EVT. We constructed a classifier based on a convolutional neural network (CNN), which was trained by CBF, Tmax, and a region of interest (ROI) mask. Compared to previous studies, to classify patients within a 4.5 h window for rt-PA thrombolysis treatment, our method is able to identify them within a 6-h window for the treatment of EVT. Meanwhile, our method has stable performance for both CTP and PWI. It means that our method enables compatible with both magnetic resonance (MR) and computed tomography (CT) devices, rather than only MR devices. Thus, our method has more potential to be used widely in hospitals, especially primary hospitals.

## Methods

### Patients

The local institutional review board approved this retrospective analysis, and the patient had signed the informed consent form. Also, patient records and images (including the source or raw imaging data) were anonymized before image analysis. Anonymized data are available on reasonable request to the corresponding author, and the data collected in the repository will be made accessible to qualified researchers worldwide, based on the recommendations of a scientific committee that will evaluate proposed research projects. The confidentiality of patients' information will be rigorously protected.

We recruited patients with AIS between April 2020 and April 2021 from the eStroke China national thrombolytic and thrombectomy imaging platform. Thirteen subcenters are registered on the platform and upload CTP or PWI images examined from patients with AIS before treatment to the eStroke platform. In addition, clinical information, including age, sex, national institute of health stroke scale (NIHSS), and exact stroke onset time are recorded. In order to align the examination performance among subcenters, we adjusted imaging protocols based on different device types, which are summarized in Table 1. To avoid the bias of the stroke onset time of patients with AIS, the data were collected by neurologists with more than 5 years of clinical experience, and they were recorded fully on the eStroke platform. Patients were recruited into this study based on the following criteria: (1) AIS due to anterior circulation artery (ACA) occlusion; (2) the recorded exact stroke onset time; (3) the recorded time of initial pretreatment imaging; (4) examined CTP or PWI before treatment; and (5) complete clinical information. All patients were anonymously recruited, and they were informed of and agreed to the study. The dataset will be released on the website https://github.com/bianyueyan/CNN-EVT.

**Table 1:** List of imaging protocols.

**CTP protocols**					
**Subcenter**	**Slice thickness (mm)**	**No. of slices**	**Total coverage (mm, cc)**	**kVp**	**mAs**
Center1	5	480	80	80	200
Center2	5	1,080	80	80	223
Center3	5	460	80	80	176
Center4	5	360	80	80	211
Center5	5	336	80	80	200
Center6	5	1,566	80	80	124
Center7	10	506	80	80	350
Center8	5	864	80	80	158
Center9	5	704	80	80	176
Center10	5	360	80	80	264
**PWI protocols**					
**Subcenter**	**Slice thickness (mm)**	**FOV (** *mm* ^2^ **)**	**Bandwidth (kHz)**	**TR/TE (ms)**	**Acquisition matrix**
Center11	5	230 × 230	28.3	1,590/32	128 × 128
Center12	5	230 × 230	31.2	1,500/19.2	96 × 128
Center13	5	230 × 230	29.4	1,740/32	128 × 128

cc, craniocaudal; mAs, milliampere-seconds; kVp, kilovoltage peak; FOV, field of view; TR, repetition time; TE, echo time.

### Experimental design

According to previous works, the stroke onset time is correlated with CBF/ADC, Tmax, and changes in the benign hypoperfusion, ischemic, and infarct core regions. These regions can be estimated by different thresholds in CBF and Tmax (9, 15). Therefore, three factors including CBF/ADC, Tmax and the region of diseased hemispheres, are correlated with the identification of the stroke onset time. In order to enable to be compatible with both CT and MR examinations, we chose CBF, Tmax and the region of diseased hemispheres as input images. In this study, we constructed three types of classifiers, namely, support vector machine (SVM), random forest (RF), and CNN, to identify patients with AIS within a 6-h window for the treatment of EVT. These classifiers were trained by three channels of images. The first channel was CBF images, the second was Tmax images, and the third was ROI mask, which was one of the regions of CBF < 30%, Tmax > 4 s, Tmax > 6 s, Tmax > 8 s, and Tmax > 10 s.

In order to compare the performance among the different classifiers (SVM, RF, and CNN), each classifier was trained by three channels of images, consisting of CBF, Tmax, and ROI masks of Tmax > 6 s. Meanwhile, for observing the differences from the ROI masks (CBF < 30%, Tmax > 4 s, Tmax > 6 s, Tmax > 8 s, and Tmax > 10 s), the CNN classifier was trained by CBF, Tmax, and each ROI mask. Through the above process, the classifier with the best performance was selected. Finally, we trained the best classifier using CBF, Tmax, and ROI mask, respectively, from CTP and PWI to compare their agreement.

### Image preprocessing

The CTP and PWI of patients with AIS were examined before the treatment, and then intra-phase rigid registration was performed to correct motion artifacts. After this, the images were smoothed using a Gaussian filter with a kernel with a width of 2.5 mm. In order to reduce disturbance of skull and cerebrospinal fluid (CSF), the images were segmented using BET2 (16) and the thresholding method, respectively, and then the ROI was selected while the rest of the image was excluded. Perfusion parameter maps, including CBF, CBV, MTT, and Tmax, were constructed by block-circulant singular value decomposition (bSVD) provided by the eStroke platform. Perfusion parameter maps were resampled to the spacing of 1 mm in the *x, y*, and *z* directions to reduce the impact of image resolutions. The resampled images were chosen as the analytical basis of feature extraction, training, and testing datasets.

According to previous studies, ROI masks segmented by different thresholds based on CBF and Tmax express the progression of AIS, which are strongly related to the stroke onset time (3–8). In order to compare their performances in estimating the stroke onset time, we segmented the ROI masks by CBF < 30%, Tmax > 4 s, Tmax > 6 s, Tmax > 8 s, and Tmax > 10 s.

### Feature extraction

Features for training machine learning methods, including SVM and RF, were generated based on CBF, Tmax images, and ROI masks, which mainly included first-order descriptive statistics, features of shape, gray level co-occurrence matrix (GLCM) features, gray level dependance matrix (GLDM) features, and gray level size zone matrix (GLSZM) features. All of the features are shown in Table 2. They were extracted with the Radiomics module in the 3D Slicer software, version 4.11 (NA-MIC, NAC, BIRN, NCIGT, and the slicer community, USA). After extracting the initial features, the principal component analysis (PCA) approach was applied to reduce dimensionality and decrease the dependance on the number of training data.

**Table 2:** List of features.

**Feature class**	**No. of features**	**Feature name**
Shape	9	Maximum 2D diameter, maximum 3D, diameter, mesh volume, minor axis length, sphericity, surface area, surface volume ratio, voxel volume
First order descriptive statistics	15	Energy, entropy, interquartile range, kurtosis, maximum, mean absolute deviation, mean, median, minimum, robust mean absolute deviation, root mean squared, skewness, total energy, uniformity, variance
GLCM	12	Autocorrelation, cluster prominence, cluster shade, cluster tendency, contrast, correlation, difference average, difference entropy, difference variance, joint average, sum entropy, sum squares
GLDM	5	Dependence entropy, dependence variance, gray level non-uniformity, gray level variance, high gray level emphasis
GLSZM	10	Gray level non-uniformity, gray level non-uniformity normalized, gray level variance, high gray level zone emphasis, large area emphasis, large area high gray level emphasis, large area low gray level emphasis, low gray level zone emphasis

### Classifier construction

We compared the performance of three types of classifiers, namely, SVM, RF, and CNN in identifying a 6-h window for the treatment of EVT. Briefly, SVM is a supervised machine learning algorithm, mainly used to process classification and regression tasks. The objective of SVM is to find a hyperplane in a N-dimensional space that is defined by the number of features in order to classify the dataset (17). RF is an ensemble learning method that can operate a variety of tasks, including regression and classification. It commonly constructs a multitude of decision trees during the training time. In a classification task, RF creates many decision trees on data samples, each of which votes based upon the results of the prediction. Finally, the output of RF means the class selected by the most trees (18). A CNN is a feed-forward neural network, which is used to handle computer vision tasks such as image classification, object detection, and image recognition (19).

**Figure 1:**
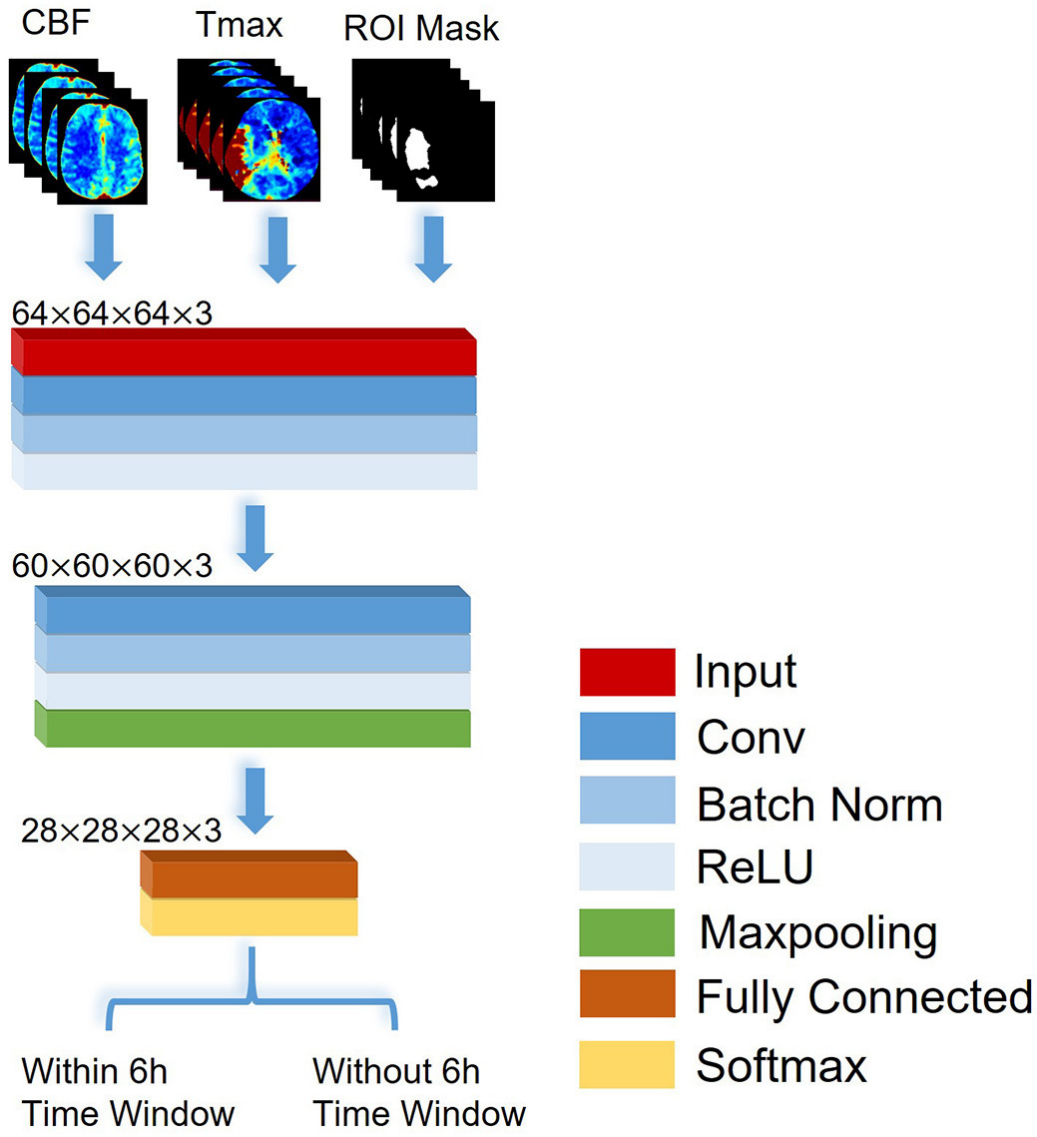
The architecture of the CNN proposed to identify patients with AIS within a 6-h window for the treatment of EVT.

In this study, a CNN was constructed based on VGGNet with 2 convolutional blocks (20), which consisted of a structure of eleven layers: an input layer, three convolutional layers, two batch normalization layers, two rectified linear unit (ReLU) layers, a max pooling layer, a fully connected layer, and a soft-max layer, which are shown in Figure 1. According to the previous works, the stroke onset time of patients with AIS was correlated with the severity and range of CBF reduction and Tmax delay. Thus, the input layer in our network was designed as a three-channel layer, which included CBF, Tmax and ROI mask respectively. The CBF and Tmax channels of the input layer can provide the detail features about the severity of CBF reduction and Tmax delay, and the ROI mask channel can present a weight map to express the range of CBF reduction and Tmax delay. The input layer was separated into blocks with the size of 64 × 64 × 64. The convolutional layer contained 16 filters with a receptive field of 5 × 5 × 5 voxels in a one-voxel stride sliding. The batch normalization layer and ReLU layer which followed the convolutional layer, batch-normalized and rectified the feature map. The max pooling layer reduced the number of rectified features, and they were flattened into a single linear vector by the fully connected layer. Finally, the classification was processed in the soft-max layer. Binary cross-entropy loss was used as loss function. Comparing VGGNet with 2 convolutional blocks, the input layer in our network included three channels, and each channel was 3D images. Apart from that, we removed a max-pooling layer in the first convolutional block in order to decrease the loss of the detail features. All classifiers were trained by fivefold cross-validation to avoid overfitting bias.

### Statistical analysis

We computed the receiver operating characteristic (ROC) curve and the area under the ROC curve (AUC), which can compare the ability of all classifiers to identify patients with AIS within a 6-h window. To determine the significance of differences among classifiers in the task of identification, we used the DeLong test to compare the AUCs of the classifiers (21). We also computed patient-wise accuracy, sensitivity, specificity, and precision for each classifier. SPSS version 22.0 (IBM, USA) and GraphPad Prism version 6.0 (GraphPad, USA) powered all of the statistical computations, with significance set at *p* < 0.05.

## Results

### Patient characteristics

We recruited 2,500 patients from the eStroke platform; 426 were excluded due to loss of original data, and 922 were excluded because of poor image quality, such as motion artifacts during scanning. Additionally, 775 with an onset time exceeding 24 h were excluded. Finally, a total of 377 patients (263 men and 114 women; mean age = 66.0 ± 11.9 years) were included in this study. The stroke onset time was 6.7 ± 5.7 h (range = 0–24 h). All patients had ACA occlusion. The patients' baseline and NIHSS are listed in Table 3.

**Table 3:** Patient characteristics.

**Characteristics**	**Values**
No. of patients	377
Age (year)	66.0 ± 11.9
Male sex^*^	263 (69.8)
Stroke onset time (h)	6.7 ± 5.7
NIHSS on admission	11.5 ± 7.2

^*^Data are the number (percentage) of patients. Except where indicated, data are mean ± SD.

### Training and testing dataset analysis

Training and testing datasets were selected randomly, which were grouped by the onset time of stroke. Table 4 shows the patient characteristics in the training and testing datasets. All *p*-values for each patient characteristic between the training and testing datasets were estimated. We observed that all *p*-values were higher than 0.05, which means that there were no significant differences in each patient characteristic between the training and testing datasets.

**Table 4:** The patient characteristics in the training and testing datasets.

**Patient characteristics**	**Training dataset**	**Testing dataset**	***P*-value**
Age (year)	66.81 ± 11.63	68.06 ± 11.64	0.3737
Sex (female/male)	98/223	16/40	0.8454
NIHSS on admission	12.08 ± 7.13	11.00 ± 7.51	0.4152
Stroke onset time (h)	5.87 ± 5.46	5.98 ± 4.60	0.0552

### Performance analysis of the classifiers

**Figure 2:**
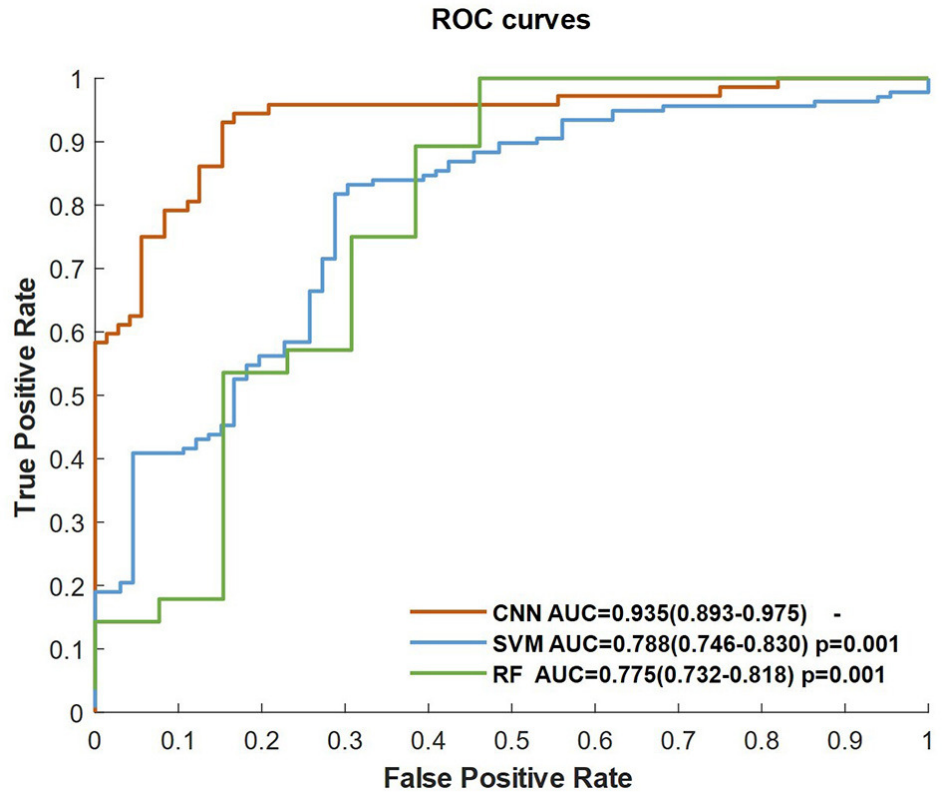
ROC curves of the classifiers, including CNN, SVM, and RF.

Figure 2 shows the ROC curves of the classifiers (SVM, RF, and CNN) for identifying patients with AIS within a 6-h treatment window for EVT. All of the AUCs of the classifiers were higher than 0.76, which was the highest AUC for identifying patients with AIS within a 4.5 h window for rt-PA thrombolysis treatment in a previous study (8). The AUC of RF was the lowest at 0.775 (0.732–0.818), while the AUC of the CNN was the highest at 0.935 (0.893–0.975). The AUC of the CNN was significantly higher than that of the SVM (*p* = 0.001) and RF (*p* = 0.001). The AUCs of the classifiers compared with the previous work are depicted in Table 5.

**Table 5:** The AUCs of classifiers of the identification of patients with AIS within a 6- and 4.5-h window.

**Classifier**	**Identifying patients within 4.5-h window**		**Identifying patients within 6-h window**
	**Ho et al**. (7)	**Kong et al**. (8)	**CBF** **+** **Tmax** **+** **ROI**
RF	0.624	0.690	0.775 (0.732–0.818)
SVM	0.669	0.746	0.788 (0.746–0.830)
CNN	–	–	**0.935 (0.893–0.975)**

Bold indicated the highest AUC for a given classifier.

### Performance analysis of the ROI masks

**Figure 3:**
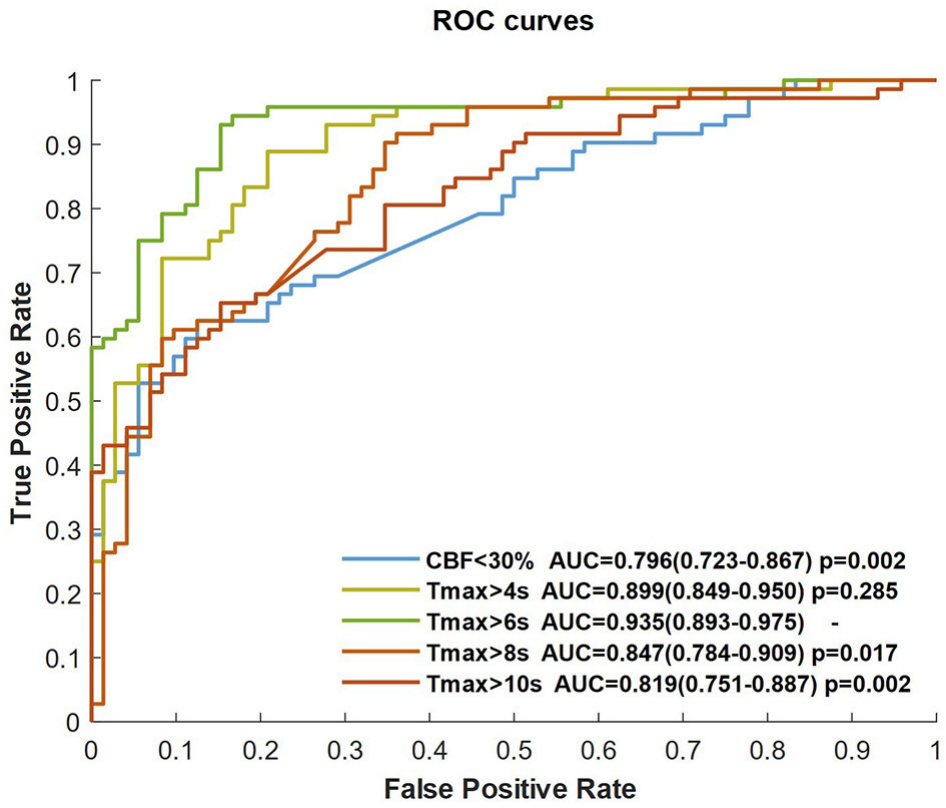
ROC curves of the CNN trained by CBF, Tmax, and each ROI mask.

The CNN classifier was trained by CBF, Tmax, and each ROI mask (respectively, CBF < 30%, Tmax > 4 s, Tmax > 6 s, Tmax > 8 s, and Tmax > 10 s), each ROC curve of which is shown in Figure 3. The AUC of Tmax > 6 s was the maximum value (AUC = 0.935), which was significantly higher than that of Tmax > 8 s and Tmax > 10 s (*p* = 0.017 and *p* = 0.002, respectively). Although the AUC of Tmax > 6 s was higher than that of Tmax > 4 s, there was no significant difference between them (*p* = 0.285). Comparing the ROI masks segmented by Tmax, the AUC of CBF < 30% was only 0.796 (0.723–0.867).

### Performance analysis of scanning devices

**Figure 4:**
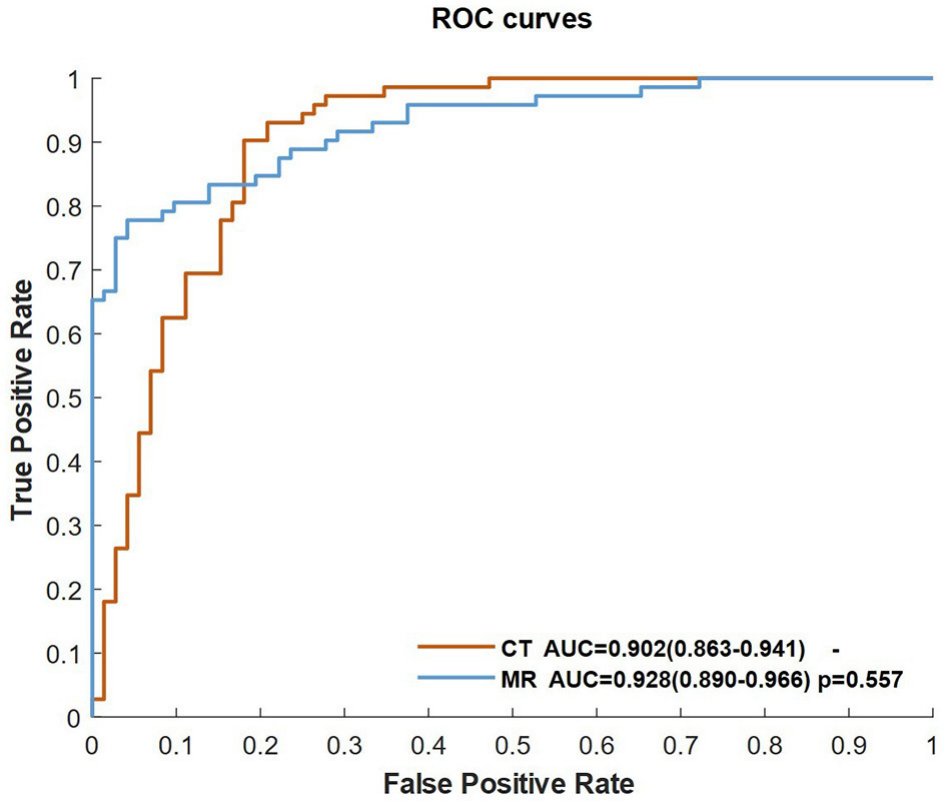
ROC curves of the classifiers of the CNN trained by CBF, Tmax, and ROI masks of Tmax > 6 s in the CT and MR groups.

We separated the training dataset into two groups (CTP and PWI), and the CNN classifier was trained by CBF, Tmax, and ROI mask of Tmax > 6 s in each group. Figure 4 shows the ROC curves of two groups. The AUCs of the two groups were higher than 0.9, and there was no significant difference between them (*p* = 0.557).

### Examples of identification

**Figure 5:**
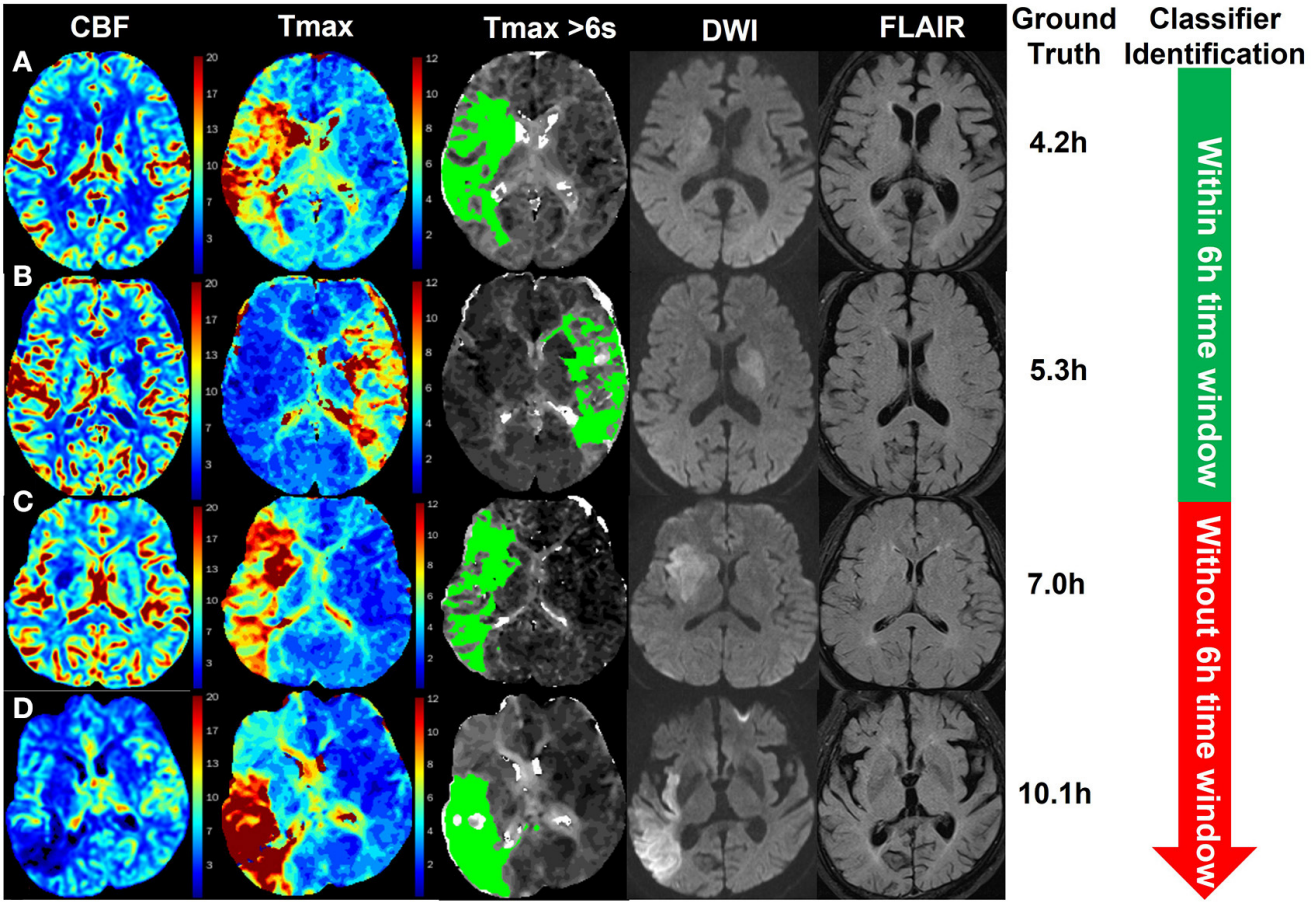
Examples of the identification of patients with AIS within a 6-h window for the treatment of EVT using the CNN classifier trained by CBF, Tmax, and ROI masks of Tmax > 6 s. The green region in the third column is the mask of Tmax > 6 s. The stroke onset times of patients **(A, B)** were, respectively, 4.2 and 5.3 h, which was identified within a 6-h time window for the treatment of EVT by the classifier. For patients **(C, D)**, their stroke onset time was 7.0 and 10.1 h, respectively, which was identified without a 6-h window by the classifier.

Figure 5 shows four examples for identifying patients with AIS within a 6-h window for the treatment of EVT using our method. The classifier was CNN-trained by CBF, Tmax, and ROI masks of Tmax > 6 s. The results of the classifier identification were matched with the ground truth, which was the accurate stroke onset time of patients. DWI and FLAIR are listed in Figure 5 for comparison with a previous study (8), which detected patients with AIS within a 4.5 h window for rt-PA thrombolysis treatment using the machine learning method and the imaging biomarker of DWI–FLAIR mismatch.

## Discussion

In this study, we proposed to use a CNN framework based on a perfusion map (CBF and Tmax) to identify patients with AIS within a 6-h window for the treatment of EVT. We compared the performance of each classifier (SVM, RF, and CNN) and differences from each ROI mask (CBF < 30%, Tmax > 4 s, Tmax > 6 s, Tmax > 8 s, and Tmax > 10 s). Our results showed that the CNN classifier trained by CBF, Tmax, and ROI masks of Tmax > 6 s had a higher performance in terms of identification within a 6-h window. Apart from this, our method had stable performance for both CTP and PWI, which means that the proposed method has higher potential to be used widely in stroke centers.

In a previous study, the progression of AIS could be directly expressed by changes in the infarct core and ischemic region (12–14). Thomalla et al. proposed that DWI–FLAIR mismatch can be deemed an imaging biomarker for identifying patients with AIS within a 4.5 h treatment window for rt-PA thrombolysis (4). Meanwhile, in the study of DIFFUSE 3, the infarct core and penumbra region could be estimated using CBF and Tmax (8). Because DWI, FLAIR, and Tmax are related to the progression of AIS, Kong at el. constructed a decoder–encoder network trained by DWI, FLAIR, and Tmax to identify patients with AIS within a 4.5 h window for rt-PA thrombolysis treatment (8). In fact, Kong's decoder–encoder network has the potential to detect this within a 6-h treatment window. However, because this network was trained only by MR examination, it was hard to be widely used in hospitals, especially primary hospitals. Thus, in order to be used for both CT and MR examination, we chose CBF and Tmax as two of the three channels of input images of classifiers instead of DWI and FLAIR, and we pulled ROI masks into the third channel of input images because their changes were correlated with the progression of AIS. This means that our method has more potential to be performed in primary hospitals.

In identifying patients with AIS within a 4.5 h window for rt-PA thrombolysis treatment, the AUC of the best classifier was 0.780 (8). The best classifier in this study was the CNN trained by CBF, Tmax, and ROI masks of Tmax > 6 s. The AUC of our method was 0.935, which is much higher than that of previous works. The reason is that the progression of AIS over time mainly influences cerebrovascular hemodynamic changes (9–11). For instance, in Figure 5, changes in CBF and Tmax had a significant relationship with the stroke onset time among patients A, B, C, and D. Although patient D was attacked by a stroke for 10.1 h, the intensity between DWI and FLAIR was not mismatched, which would have been misestimated in previous works. Apart from this, our results showed that the CNN has a stronger ability to capture hidden features and signal changes from CBF and Tmax, compared to machine learning methods such as SVM and RF. Moreover, by comparing the performance of classifiers trained by different ROI masks, our results showed that the AUC of Tmax > 6 s was the highest in all ROI masks, although it was not significantly higher than that of Tmax > 4 s (*p* = 0.285). According to a previous work (9), the region of Tmax > 6 s includes an infarct core and penumbra, while the regions of CBF < 30% and Tmax > 10 s only include an infarct core, and the region of Tmax > 8 s includes an infarct core and a part of penumbra. For the region of Tmax > 4 s, it includes benign hypoperfusion, an infarct core and penumbra, which should include more features than that of Tmax > 6 s, but the benign hypoperfusion in the region of Tmax > 4 s is always misestimated because of personalizing. For example, Tmax values in the deep area of white matter without lesions are commonly more than 4 s for patients with AIS. Therefore, we recommend the CNN classifier trained by CBF, Tmax, and ROI masks of Tmax > 6 s rather than Tmax > 4 s.

This study has some methodological limitations that need to be addressed. First, the sample size was relatively lower than that of other studies based on deep learning algorithms. However, data were collected from 13 centers, with eight types of CT and MR scanners, uniformly distributed between 0 and 24 h from the stroke onset time. Thus, the sample size was enough to support the training of the CNN model in this study. Second, data were collected retrospectively, and some inaccurate information was involved. In fact, a prospective study to evaluate the performance of our method in clinical use is a future avenue for investigation, but it does not enable to assume the clinical potential of this study. In the future, a larger, randomized, and prospective study will be designed to evaluate the performance of this method.

## Conclusion

In this study, a CNN classifier trained by CBF, Tmax, and ROI masks of Tmax > 6 s, has good performance to identify patients with AIS within a 6-h window for the treatment of EVT. Comparing with existing works to classify patients within a 4.5-h window for the treatment of rt-PA thrombolysis, to the best of our knowledge, this is the first work to assist the treatment of EVT. Meanwhile, our method performs the identifying task using CBF and Tmax, which can be acquired by CTP or PWI. It means that our method is compatible with both CT and MR devices, while previous works only support MR devices because their inputs rely on DWI and FLAIR images which are examined only by MR devices. Commonly, CT examination is faster than MR, which benefits to bring the patients out of danger. Therefore, it has the potential to be widely used to accurately estimate the stroke onset time of patients with WUS.

## Data availability statement

The original contributions presented in the study are included in the article/supplementary material, further inquiries can be directed to the corresponding authors.

## Ethics statement

Ethical review and approval was not required for the study on human participants in accordance with the local legislation and institutional requirements. Written informed consent from the [patients/ participants OR patients/participants legal guardian/next of kin] was not required to participate in this study in accordance with the national legislation and the institutional requirements.

## Author contributions

HG and YB designed the study. HG, YC, and JW collected the data. HG, YB, and HY were involved in the interpretation of data. YB, HZ, and GC analyzed and visualized the data. HG and YB drafted the manuscript. QY and LW revised the manuscript. All authors read and approved the final manuscript.
